# In Vitro Effects of *Centaurea ovina* (Roudbari Cornflower) Aqueous and Ethanolic Extracts on *Staphylococcus saprophyticus*: Growth Inhibition, Antibiofilm Activity, and Reduced *ureC*/*uafA* Transcripts

**DOI:** 10.1155/ijm/7908693

**Published:** 2026-07-15

**Authors:** Sara Sadeghi Keleshteri, Mohammad Javad Mehdipour Moghaddam, Akbar Norastehnia

**Affiliations:** ^1^ Department of Biology, University Campus 2, University of Guilan, Rasht, Iran, guilan.ac.ir; ^2^ Department of Biology, Faculty of Sciences, University of Guilan, Rasht, Iran, guilan.ac.ir

**Keywords:** antimicrobial, biofilm, *Centaurea ovina*, qRT-PCR, *Staphylococcus saprophyticus*, virulence

## Abstract

**Objectives:**

*Staphylococcus saprophyticus* frequently causes community‐acquired urinary tract infections (UTIs) and is characterized by multidrug resistance (MDR) and biofilm formation. This study evaluated the in vitro antibacterial and antibiofilm effects of aqueous and ethanolic extracts of *Centaurea ovina* (Roudbari cornflower) against clinical *S. saprophyticus* isolates and measured changes in *ureC* and *uafA* transcript levels after exposure to the extracts.

**Methods:**

The aqueous and 80% ethanolic extracts of *C. ovina* were analyzed for total phenolics, flavonoids, flavonols, anthocyanins, and DPPH radical‐scavenging activity. Antibacterial activity against three clinical isolates (SS1–SS3) was assessed using agar well diffusion and broth microdilution (MIC/MBC). Biofilm inhibition was measured by crystal violet staining. In the MDR isolate (SS2), *ureC* and *uafA* transcript levels were measured by SYBR Green qRT‐PCR (2−*ΔΔ*Ct) after treatment with the ethanolic extract at a sub‐MIC concentration (1/2MIC), with results normalized to 16S rRNA.

**Results:**

The ethanolic extract contained more flavonoids and flavonols and exhibited stronger DPPH scavenging activity than the aqueous extract, which had more total phenolics. In broth microdilution, the ethanolic extract inhibited SS1–SS3 growth at 48 mg/mL (MIC), whereas the aqueous extract did not reach an MIC within the tested range (MIC > 48 mg/mL). No bactericidal effect was observed for the ethanolic extract (MBC > 48 mg/mL). Both extracts reduced biofilm inhibition in a concentration‐dependent manner. The ethanolic extract demonstrated significantly higher efficacy, achieving up to 85.6% inhibition (for SS2) at 48 mg/mL, compared with the aqueous extract, which reached up to 71.6% inhibition (for SS2) at the same concentration. In SS2, the ethanolic extract at the sub‐MIC reduced *ureC* and *uafA* transcript levels to 0.278 and 0.272 times those of untreated controls, respectively.

**Conclusions:**

Both aqueous and ethanolic *C. ovina* extracts showed in vitro growth‐inhibitory and antibiofilm activity against clinical *S. saprophyticus* isolates, with the ethanolic extract being more effective. This effect was associated with lower expression of two uropathogenesis‐related genes in an MDR isolate. Further chemical analysis, testing at lower concentrations, and in vivo studies are needed before clinical use.

## 1. Introduction

Antibiotics have greatly reduced morbidity and mortality from bacterial infections; however, the global rise in antimicrobial resistance (AMR) has diminished their effectiveness and now represents a significant public health concern, much like other complex pathological conditions requiring clinical observation [1–4]. This challenge has increased interest in alternative or adjunctive antimicrobial strategies, including bioactive compounds derived from medicinal plants. Plant secondary metabolites, particularly polyphenols and flavonoids, have demonstrated antibacterial and antibiofilm activities and may enhance the efficacy of conventional antibiotics [5–8]. Urinary tract infections (UTIs) are among the most prevalent bacterial infections, particularly in women, and are frequently managed empirically rather than guided by laboratory results [9–11]. Although nitrofurantoin and fosfomycin are recommended as first‐line therapies for uncomplicated cystitis, local resistance patterns and specific bacterial characteristics can complicate treatment [12, 13]. *Staphylococcus saprophyticus* is an important cause of community‐acquired UTIs, particularly in young, sexually active women [14]. Its pathogenicity is associated with adhesion to uroepithelial cells, urease activity, and biofilm formation, all of which may contribute to infection persistence and recurrence [15–17]. The uro‐adherence factor A gene (*uafA*) is involved in bacterial attachment, whereas urease‐associated genes such as *ureC* facilitate urea hydrolysis and are linked to uropathogenesis [15–17]. Therefore, interventions that inhibit bacterial growth, biofilm formation, or the expression of uropathogenesis‐associated genes are of considerable interest.

The *Centaurea* genus includes species known for antimicrobial and antioxidant properties, primarily attributed to their phenolic and flavonoid constituents [18–20]. The choice of extraction solvent significantly influences the spectrum of compounds obtained, with hydroethanolic mixtures often yielding a broader range of bioactive compounds than water alone [21–23]. *Centaurea ovina* (Roudbari cornflower) is one such species [24]; however, limited data exist on the effects of its extracts on clinical *S. saprophyticus* isolates, particularly regarding biofilm inhibition and modulation of disease‐associated gene expression.

Accordingly, this study evaluated the in vitro antibacterial and antibiofilm activities of aqueous and ethanolic *C. ovina* extracts against clinical *S. saprophyticus* isolates and assessed changes in *ureC* and *uafA* transcript levels in an MDR isolate after treatment with the extracts.

## 2. Materials and Methods

### 2.1. Collection of Plant Samples

*C. ovina* (Roudbari cornflower) was collected from the Rudbar region of Gilan Province, Iran, at geographical coordinates N 36°50 ^′^17.35 ^″^ and E 49°27 ^′^35.79 ^″^. After collection, the plant was thoroughly washed to remove environmental contamination and transferred to the plant physiology laboratory for further processing. The plant material was left in an open‐air environment for 5 days to partially dry. It was then dried completely in an oven at 40°C for 48 h. After drying, the plant was ground using a Moulinex grinder and sieved through a 500‐micron mesh to obtain a fine powder. The dried powder was stored in a desiccator until further use to prevent moisture [24].

### 2.2. Preparation of Aqueous and Ethanolic Extracts

Dried aerial parts of *C. ovina* were powdered and used for extraction. For ethanolic extraction, 5 g of dried powder was mixed with 50 mL of 80% (*v*/*v*) ethanol in a sealed flask. The mixture was agitated in an incubator shaker (100 rpm) at 25°C for 72 h. It was then centrifuged (8000 rpm, 10 min, 4°C), and the supernatant was collected. The solvent was removed from the supernatant by concentrating it at 45°C until dry. The dried residue was stored in a desiccator until use. For aqueous extraction, the same procedure was followed, using distilled water instead of 80% ethanol [21–23].

For microbiological assays, extract solutions were filter‐sterilized using 0.45‐*μ*m membrane filters; for molecular assays (RNA/qRT‐PCR), solutions were filter‐sterilized using 0.22‐*μ*m membrane filters.

To prepare working solutions, a 48 mg/mL stock was prepared by dissolving 0.12 g of dried extract in 2.5 mL of solvent (distilled water for the aqueous extract and 5% DMSO for the ethanolic extract). Two‐fold serial dilutions were prepared to obtain final concentrations of 48, 24, 12, and 6 mg/mL for antimicrobial and antibiofilm assays. Solvent controls contained the corresponding solvent at the same final concentration as the test wells.

### 2.3. Secondary Metabolite Quantification

The secondary metabolites and antioxidant activity of the plant extracts were quantified using various spectrophotometric methods. All plant extracts were prepared at a stock concentration of 100 mg/mL (1 g of dried plant powder in 10 mL of solvent). The experimental procedures are outlined below.

#### 2.3.1. Phenols

The total phenolic content was determined using the Folin–Ciocalteu method. One milliliter of the plant extract was mixed with 2 mL of Folin–Ciocalteu reagent, followed by the addition of 2 mL of 20% sodium carbonate solution. The mixture was incubated at room temperature for 30 min, and absorbance was measured at 765 nm [25–27].

#### 2.3.2. Flavonoids

The total flavonoid content was determined using the aluminum chloride colorimetric method described by Lamaison et al. (1991). Briefly, 1 mL of plant extract was mixed with 1 mL of 2% aluminum chloride solution. After 10 min, absorbance was measured at 510 nm. The flavonoid content was calculated using a standard quercetin curve [28].

#### 2.3.3. Flavonols

The total flavonol content was measured using the method of Nagai et al. (2007). One milliliter of plant extract was mixed with 1 mL of 5% sodium nitrite, followed by the addition of 1 mL of 10% aluminum chloride and 2 mL of 1 M sodium hydroxide. Absorbance was measured at 360 nm, and the flavonol content was expressed as mg of quercetin equivalents per gram of extract [28].

#### 2.3.4. Anthocyanins

The total anthocyanin content was determined using the pH differential method. One milliliter of plant extract was mixed with 3 mL of buffer solutions at pH1.0 and pH4.5. Absorbance was measured at 520 nm, and the anthocyanin content was calculated from the difference in absorbance between the two pH values [29, 30].

#### 2.3.5. Antioxidant Activity (DPPH)

The free radical scavenging activity was determined using the DPPH method. The plant extracts were prepared at a stock concentration of 100 mg/mL (as detailed in Section 2.3). Briefly, 50 *μ*L of the extract was added to 950 *μ*L of a 0.1 mM DPPH solution in ethanol, achieving a final concentration of 5 mg/mL in the reaction mixture. The mixture was incubated in the dark for 30 min at room temperature. The absorbance was measured at 517 nm against a blank. The percentage of DPPH radical scavenging activity was calculated using the following formula:

DPPH activity%=A0−A1A0×100,

where A_0_ is the absorbance of DPPH without the sample, and A_1_ is the absorbance in the presence of the sample [31].

### 2.4. Bacterial Strain Identification

*S. saprophyticus* isolates were isolated from urine samples of patients with UTIs. The bacterial species were identified using both biochemical and molecular methods. Biochemical identification was performed using standard differential tests, including catalase, coagulase, and novobiocin resistance, which differentiate *S. saprophyticus* from other staphylococcal species [32]. Molecular identification was performed by amplifying the 16S rRNA gene and the virulence‐related genes *uafA* and *ureC* using specific primers. These molecular methods were used to further confirm the bacterial species and to detect key virulence factors associated with *S. saprophyticus*.

### 2.5. Antimicrobial Activity Assays

#### 2.5.1. Well Diffusion Method

A 0.5 McFarland suspension of each isolate (≈1.5 × 10^8^ CFU/mL) was prepared and uniformly swabbed onto Mueller–Hinton agar plates. Wells (6 mm in diameter) were punched aseptically, and 30 *μ*L of each extract concentration (48, 24, 12, and 6 mg/mL) was added to each well. Distilled water (aqueous extract solvent) and 5% DMSO (ethanolic extract solvent) served as negative/solvent controls. A standard antibiotic disc active against *S. saprophyticus* was included as a positive control. Plates were incubated at 37°C for 24 h, and inhibition zones were measured in millimeters [33].

#### 2.5.2. Microdilution Method (MIC and MBC)

MIC and MBC were determined by broth microdilution in sterile 96‐well microtiter plates. Bacterial suspensions were adjusted to 0.5 McFarland and diluted in Mueller–Hinton broth (MHB) to yield a final inoculum of approximately 5 × 10^5^ CFU/mL per well. Extracts were tested at final concentrations of 48, 24, 12, and 6 mg/mL (twofold serial dilutions). Growth control wells contained MHB plus inoculum without extract. Sterility control wells contained MHB only. Solvent control wells contained MHB with the corresponding solvent (distilled water or 5% DMSO) plus inoculum. Extract background wells contained MHB plus extract without inoculum to allow blank correction. Plates were incubated at 37°C for 24 h under static conditions, and growth was assessed by measuring OD at 630 nm after appropriate blank correction [33]. MIC was defined as the lowest concentration at which no detectable growth was observed relative to the growth control. For MBC determination, aliquots from wells containing concentrations at or above the MIC were subcultured onto Mueller–Hinton agar and incubated at 37°C for 24 h; MBC was defined as the lowest concentration yielding no colony growth [33].

### 2.6. Biofilm Inhibition Assay

Biofilm inhibition was assessed using the crystal violet method in 96‐well microtiter plates. Briefly, bacterial cultures were incubated with extract concentrations (48, 24, 12, and 6 mg/mL) at 37°C for 48 h. After incubation, wells were gently washed with distilled water to remove planktonic cells. Adherent biofilms were stained with 1% (*w*/*v*) crystal violet, and excess stain was removed by washing. The bound dye was solubilized with 30% (*v*/*v*) acetic acid, and absorbance was measured at 570 nm [34]. Biofilm inhibition was calculated as follows:

Biofilm inhibition %=ODcontrol−ODsample/ODcontrol×100.

Appropriate solvent controls were included and processed identically.

### 2.7. Extraction of Bacterial DNA and PCR Detection of Virulence Genes

Bacterial DNA was extracted using the boiling method. After activation of the bacterial culture, a bacterial suspension was prepared to match the 0.5 McFarland standard and incubated. The suspension was heated to 100°C for 4–5 min to lyse the bacteria and release the DNA, which was then used for subsequent gene detection.

For PCR amplification, specific primers for the 16S rRNA gene, *uafA*, and *ureC* were used. The 16S rRNA gene served as an internal control, whereas *uafA* and *ureC* are virulence‐related genes of *S. saprophyticus*. Primer sequences and details for these genes are listed in Table 1. The PCR products were analyzed by agarose gel electrophoresis, and the presence of the corresponding bands supported the molecular identification of the bacterial isolate and the detection of the virulence genes.

**Table 1:** Primers used for PCR amplification of *S. saprophyticus* virulence genes.

GC content (%)	Melting temperature (TM)	Primer sequence (bp)	Direction	Target gene
55	59/35	AGTGAGAATGCAGCGGTGTGAG	Forward	16S rRNA
55	59/35	TGTATTCATCCATGCCTACGCC	Reverse	16S rRNA
55	60/25	GTAGATGATCGTGTTGGTGAAG	Forward	*uafA*
55	57/87	AGGCATGTTTCCCTCCATTAGG	Reverse	*uafA*
50	57/30	CCATGTAATAAGTCGTGGGTT	Forward	*ureC*
50	57/30	GCAAGTTCATCCAGCTGATGA	Reverse	*ureC*

The PCR amplification was carried out under the following thermal cycling conditions: an initial denaturation at 95°C for 15 min, followed by 35 cycles of denaturation at 95°C for 15–20 s, primer annealing at 60°C for 20 s, and extension at 72°C for 20 s, with a final extension step at 72°C for 5 min.

### 2.8. RNA Extraction and cDNA Synthesis

Total RNA was extracted from untreated and plant extract‐treated *S. saprophyticus* cultures using the RNX‐Plus reagent (CinnaGen, Iran), following the manufacturer′s instructions. After 24 h of treatment, the bacterial cells were harvested by centrifugation at 10,000 × g for 10 min at 4°C. The RNA pellets were resuspended in RNase‐free water and stored at −80°C for future analysis. The quantity and purity of the extracted RNA were assessed using a NanoDrop spectrophotometer (Thermo Fisher Scientific, United States). Only RNA samples with A260/280 ratios between 1.8 and 2.0 were considered suitable for downstream applications. RNA integrity was further confirmed by running the samples on a 1% agarose gel to assess degradation. Intact RNA should produce clear, sharp bands, indicating high‐quality extraction.

For first‐strand cDNA synthesis, 1 *μ*g of total RNA was reverse‐transcribed using the RevertAid First Strand cDNA Synthesis Kit (Thermo Fisher Scientific, United States) and random hexamers. The reaction was performed in a 20 *μ*L total volume.

### 2.9. Gene Expression Analysis (Real‐Time PCR)

qRT‐PCR was performed using SYBR Green Master Mix (Amplicon, Denmark) on a StepOnePlus Real‐Time PCR System (Thermo Fisher Scientific, United States). Each 20 *μ*L reaction contained 10 *μ*L SYBR Green Master Mix (2x), 1 *μ*L forward primer (10 *μ*M), 1 *μ*L reverse primer (10 *μ*M), 2 *μ*L of 1:10‐diluted cDNA, and 6 *μ*L nuclease‐free water. Real‐time PCR amplification was performed with an initial denaturation at 95°C for 10 min, followed by 40 cycles of denaturation at 95°C for 15–30 s, primer annealing at 60°C for 30 s, and extension at 72°C for 30 s, with a final extension step at 72°C for 10 min. The specificity of amplification was confirmed by melt curve analysis over a temperature range of 65°C–95°C.

Relative expression levels of the *uafA* and *ureC* genes were normalized to 16S rRNA as the reference gene. Primers were designed using Primer‐BLAST (NCBI) and validated for specificity and amplification efficiency (90%–110%). Gene expression was calculated using the 2^−*ΔΔ*Ct^ method [35–37]. All reactions were performed in technical triplicates, and no‐template controls (NTCs) were included to monitor for contamination [38].

### 2.10. Statistical Analysis

Data are presented as mean ± SD from at least three independent measurements. Comparisons between groups were performed using one‐way ANOVA (GraphPad Prism, Version 9), and *p* < 0.05 was considered statistically significant.

## 3. Results

**Figure 1:**
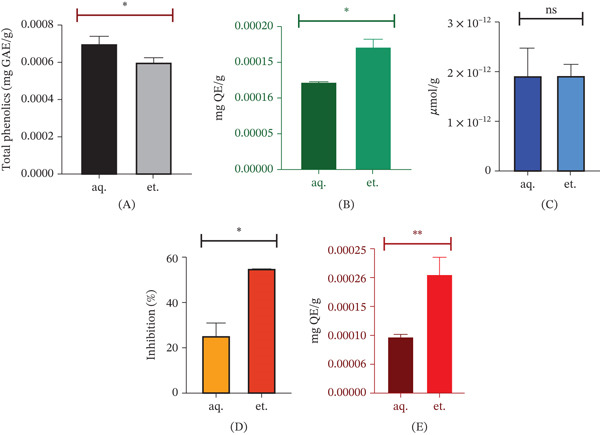
(A) Comparison of total phenolic content (mg GAE/g), (B) total flavonoid content (mg QE/g extract), (C) anthocyanin content (*μ*mol/g), (D) DPPH radical scavenging activity (% inhibition), and (E) total flavonol content (mg QE/g) in aqueous and ethanolic extracts of *C. ovina*. Data are presented as mean ± SD. The asterisks indicate statistical significance at the *p* < 0.05 level and "ns" denotes not significant.

The secondary metabolites in *C. ovina* extracts, including flavonoids, flavonols, anthocyanins, total phenolics, and antioxidant activity, were quantitatively analyzed (Figure 1). The total phenolic content was found to be significantly higher in the aqueous extract (0.338 ± 0.024 mg GAE/g) compared with the ethanolic extract (0.289 ± 0.010 mg GAE/g; *p* < 0.05). Conversely, the ethanolic extract demonstrated significantly higher levels of total flavonoids (0.0599 ± 0.0006 mg QE/g) and flavonols (0.1020 ± 0.0141 mg QE/g) than the aqueous extract (0.0486 ± 0.0020 mg QE/g for both; *p* < 0.05). Although no significant difference was observed in anthocyanin content between the two extracts (*p* = 0.2), the antioxidant activity, as assessed by the DPPH radical scavenging assay, was significantly higher in the ethanolic extract compared with the aqueous counterpart (*p* = 0.01).

The SS1–SS3 isolates were identified using standard biochemical tests. All isolates were catalase‐positive and coagulase‐negative, consistent with *S. saprophyticus*. Resistance to novobiocin was confirmed in all strains, further supporting the species identification.

Antibiotic susceptibility testing showed that SS1 was susceptible to most antibiotics, except penicillin (Table 2). SS2 was resistant to penicillin, azithromycin, erythromycin, and clindamycin, while remaining susceptible to tetracycline, nitrofurantoin, cefoxitin, doxycycline, and minocycline. SS3 was resistant only to penicillin and susceptible to tetracycline, nitrofurantoin, erythromycin, clindamycin, cefoxitin, minocycline, and ciprofloxacin. Overall, tetracycline, nitrofurantoin, doxycycline, minocycline, rifampin, and ciprofloxacin were effective, whereas penicillin, azithromycin, erythromycin, and clindamycin showed the highest resistance. Based on its resistance profile, SS2 was classified as an MDR isolate.

**Table 2:** Antibiotic sensitivity and resistance profiles of *S. saprophyticus* strains (SS1, SS2, and SS3) against multiple antibiotics.

Strain	Gender	Age	Source	Antibiotic pattern	
SS1	Female	20	Urine	Sensitive	Resistance
				Tetracycline	Penicillin
				Azithromycin	
				Cotrimoxazole	
				Ciprofloxacin	
				Clarithromycin	
				Nitrofurantoin	
				Oxacillin	
				Doxycycline	
				Clindamycin	
				Erythromycin	
				Rifampin	
				Cefoxitin	
				Minocycline	

SS2	Female	30	Urine	Cotrimoxazole	Penicillin
				Nitrofurantoin	Azithromycin
				Cefoxitin	Erythromycin
				Tetracycline	Clindamycin
				Clarithromycin	
				Oxacillin	
				Ciprofloxacin	
				Doxycycline	
				Minocycline	
				Rifampin	

SS3	Female	51	Urine	Tetracycline	Penicillin
				Nitrofurantoin	
				Erythromycin	
				Clindamycin	
				Cefoxitin	
				Minocycline	
				Cotrimoxazole	
				Doxycycline	
				Azithromycin	
				Rifampin	
				Oxacillin	
				Clarithromycin	
				Ciprofloxacin	

**Figure 2:**
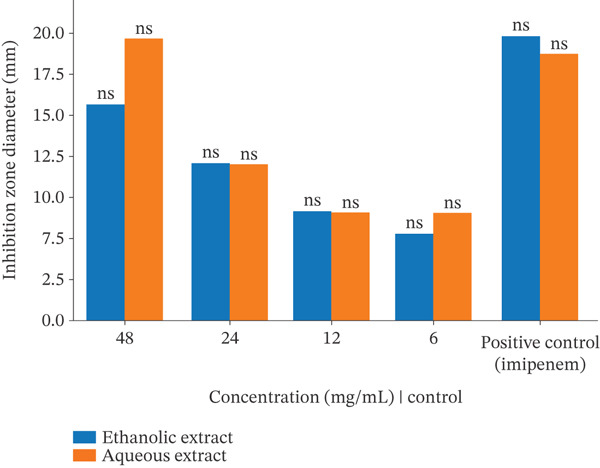
Antibacterial activity of ethanolic and aqueous extracts of *C. ovina* against the SS2 isolate evaluated by agar well diffusion assay. Inhibition zone diameters (mm) are shown for extract concentrations of 48, 24, 12, and 6 mg/mL. Imipenem was used as a positive control and 5% DMSO as a negative control. Differences between ethanolic and aqueous extracts at each concentration were not statistically significant (ns).

In the agar well diffusion assay, both the ethanolic and aqueous extracts were tested at four concentrations (48, 24, 12, and 6 mg/mL) against SS2. The results, averaged across three replicates, are shown in Figure 2. At the highest concentration (48 mg/mL), the aqueous extract produced a zone of inhibition of 20 mm, whereas the ethanolic extract produced a zone of 15 mm. This difference was observed primarily at the highest concentration; at lower concentrations (12 and 6 mg/mL), both extracts showed comparable inhibitory activity, with no meaningful difference. The imipenem positive control produced the largest inhibition zone (20 mm), whereas the DMSO solvent control showed no inhibitory activity.

In the broth microdilution assay, the ethanolic extract exhibited a MIC of 48 mg/mL against all three isolates (SS1, SS2, and SS3), indicating inhibition of bacterial growth within the tested concentration range. In contrast, the aqueous extract showed no inhibitory effect at the tested concentrations (MIC > 48 mg/mL). Furthermore, no MBC endpoint was observed for the ethanolic extract at the tested concentrations (MBC > 48 mg/mL), suggesting that the extract exerted a bacteriostatic rather than bactericidal effect under the experimental conditions.

**Figure 3:**
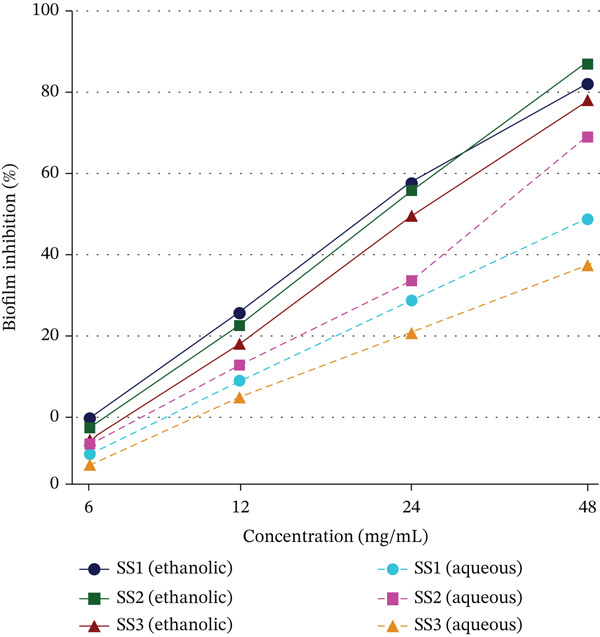
Biofilm inhibition percentage of clinical isolates (SS1, SS2, and SS3) treated with varying concentrations of ethanolic and aqueous extracts (6–48 mg/mL). The data points represent the mean values, illustrating a dose‐dependent increase in antibiofilm activity for both extract types across all tested strains. The ethanolic extract showed a higher inhibition rate compared with the aqueous extract, particularly at the maximum concentration.

The antibiofilm activity of the ethanolic and aqueous extracts against the clinical isolates (SS1, SS2, and SS3) is presented in Figure 3. Both extracts demonstrated a concentration‐dependent inhibitory effect, with efficacy increasing significantly as concentrations rose from 6 to 48 mg/mL. The ethanolic extract exhibited stronger antibiofilm potential compared with the aqueous extract across all tested isolates. At the maximum concentration (48 mg/mL), the ethanolic extract achieved inhibition rates of up to 85.6%, whereas the aqueous extract showed a maximum inhibition of approximately 71.6%. Among the tested isolates, SS2 displayed the highest susceptibility to the treatments, particularly when exposed to the ethanolic extract at the highest dose.

**Figure 4:**
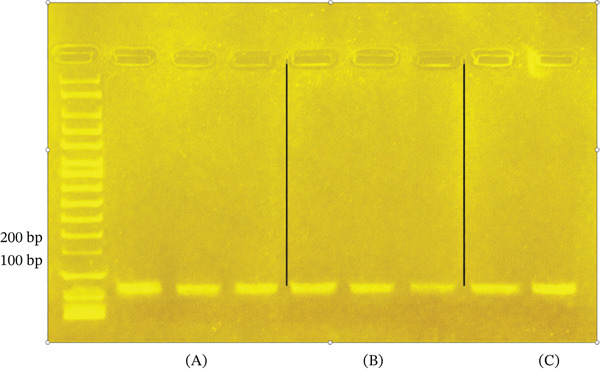
Agarose gel electrophoresis of PCR products for virulence‐associated genes in the SS2 isolate. (A) PCR amplification of the *ureC* gene (122 bp). (B) PCR amplification of the *uafA* gene (125 bp). (C) PCR amplification of the 16S rRNA (140 bp) gene was used as an internal control. PCR reactions were performed at different annealing temperatures, and the optimal temperatures yielding the most specific bands were selected for subsequent analyses.

PCR amplification of virulence‐associated genes was performed for SS2. PCR products for *ureC* (122 bp), *uafA* (125 bp), and 16S rRNA (internal control) were successfully amplified, as confirmed by agarose gel electrophoresis (Figure 4), indicating the presence of these targets in SS2.

**Figure 5:**
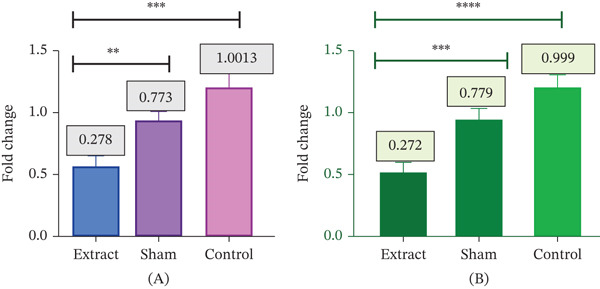
Fold change analysis of *ureC* (left) and *uafA* (right) gene expression in SS2 isolate treated with ethanolic extract at sub‐MIC concentration, sham, and control. Significant downregulation of both genes was observed in the extract‐treated groups compared with the control, as indicated by asterisks (*p* < 0.05).

In SS2, exposure to the ethanolic extract at the sub‐MIC significantly reduced *ureC* and *uafA* transcript levels relative to the untreated control (Figure 5). Expression decreased to 0.278‐fold (≈72.3%) for *ureC* and 0.272‐fold (≈72.8%) for *uafA* (*p* < 0.05). The solvent control (5% DMSO) showed smaller reductions (0.773‐fold for *ureC* and 0.779‐fold for *uafA*). Melt curve analysis showed single, sharp peaks for each primer pair (16S rRNA ~77°C–78°C; *ureC*/*uafA* ~85°C–86°C), supporting amplification specificity and the absence of primer dimers or nonspecific products.

## 4. Discussion

This study investigated the effects of aqueous and ethanolic extracts of *C. ovina* on clinical *S. saprophyticus* isolates, with an emphasis on growth inhibition, biofilm suppression, and modulation of virulence‐associated gene transcripts. The ethanolic extract showed measurable growth inhibition in broth (MIC within the tested range), exhibited stronger antibiofilm effects than the aqueous extract, and was associated with reduced *ureC* and *uafA* transcript levels in the MDR isolate SS2.

The reason for investigating plant‐derived antimicrobials is reinforced by the increasing threat of AMR and the urgent need for alternative or supplementary strategies to maintain treatment efficacy [2–4]. Plant secondary metabolites, particularly polyphenols and flavonoids, have demonstrated antibacterial activity and may synergize with standard antibiotics, thereby encouraging ongoing research into medicinal plants as sources of active compounds [5–8]. In this study, the differences seen between aqueous and ethanolic extracts probably result from solvent‐dependent phytochemical extraction, since the polarity of the extraction solvent significantly impacts the chemical profile obtained from plant materials [21–23].

Antibacterial activity was evaluated using both agar well diffusion and broth microdilution assays. These techniques may give different results for crude extracts because inhibition zones depend on diffusibility and interactions with the agar, whereas microdilution more directly measures growth inhibition in liquid media, making it preferable for determining MIC values [33]. In our study, the aqueous extract showed a larger inhibition zone than the ethanolic extract at the highest tested concentration in diffusion assays, but only the ethanolic extract had an MIC within the tested range in broth microdilution. This discrepancy can happen when diffusible components do not sustain the same inhibitory effect in broth due to solubility issues, precipitation, or interactions with broth components. However, in cases of high turbidity and extract precipitation under static incubation, visual differentiation between bacterial growth and extract‐related signals remains challenging [33]. It is important to note that although the observed MIC of 48 mg/mL is relatively high compared with pure antibiotics, it is consistent with the reported activity of other crude extracts from the *Centaurea* genus against *Staphylococcus* species [39–41]. Given that crude extracts consist of complex phytochemical mixtures, such values are frequently reported and serve as a promising baseline for future bioassay‐guided fractionation to isolate more potent active constituents. Since crude extracts can cause turbidity or coloration affecting OD‐based readings, including extract‐only background controls (blank correction) is crucial for accurate interpretation [33].

From a pharmacodynamic perspective, the absence of an MBC endpoint for the ethanolic extract within the tested concentration range indicates a predominantly bacteriostatic effect under these conditions. Although bactericidal activity is often preferred, bacteriostatic agents may retain clinical utility, especially if they also reduce biofilm formation and virulence‐associated traits. Further validation is required to identify the active constituents and assess their potency [5–8].

In our assays, both extracts inhibited biofilm formation across all isolates in a dose‐dependent manner, with the ethanolic extract showing markedly stronger efficacy. At the highest concentration (48 mg/mL), the ethanolic extract achieved an inhibition rate of up to 85.6%, whereas the aqueous extract reached a maximum of approximately 71.6%. This robust antibiofilm activity, especially at the MIC and sub‐MIC levels, is particularly relevant for *S. saprophyticus*, as its capacity for adhesion is a prerequisite for urinary tract colonization [15–17]. Plant phenolics and flavonoids, which were found in higher concentrations in our ethanolic extract, are known to interfere with early bacterial adhesion and extracellular matrix production [5–8, 42–44]. The superior performance of the ethanolic extract in both growth inhibition and biofilm suppression suggests that its phytochemical constituents may have a synergistic effect on both cell viability and surface attachment mechanisms.

A key aim of this study was to evaluate virulence‐associated gene expression in response to exposure to the plant extracts. The pathogenicity of *S. saprophyticus* is linked to urothelial adhesion and urease activity [15–17]. The *uafA* gene encodes a uro‐adherence factor implicated in attachment, whereas *ureC* contributes to urea hydrolysis and persistence. In the MDR isolate SS2, exposure to the ethanolic extract at a sub‐MIC was associated with significant downregulation of *ureC* and *uafA*. The significant reduction in biofilm biomass by the ethanolic extract is consistent with the downregulation of *uafA* (a major adhesin) and *ureC* observed in our qRT‐PCR analysis, suggesting that the extract targets biofilm formation at the transcriptional level. Since gene expression analysis was performed at a concentration that did not inhibit bacterial growth, these changes suggest a targeted antivirulence effect rather than general growth suppression.

Nevertheless, transcriptional modulation alone does not definitively confirm antivirulence activity. Additional studies are required to further validate these findings, including assessment of gene expression across multiple subinhibitory concentrations and the use of functional phenotypic assays such as urease activity measurements and adhesion assays to uroepithelial cells [15–17, 35–38].

Several limitations should be considered. The study included only three isolates for MIC and biofilm assays, and diffusion and gene‐expression analyses were restricted to a single isolate (SS2). In addition, molecular identification of the clinical isolates was based on PCR amplification of the 16S rRNA, *uafA*, and *ureC* genes, without sequencing‐based confirmation. Although these markers provide supportive evidence for identifying *S. saprophyticus*, sequencing would be required for definitive species‐level confirmation. Chemical characterization of the extracts was not conducted; chromatographic profiling (HPLC or LC–MS) would facilitate identification of active constituents [20–22]. Furthermore, the DPPH assay lacked a standard positive control, and although qRT‐PCR followed established protocols, explicit reporting of biological replicates would strengthen confidence in the results [35–38]. Additionally, although image‐based analysis of crystal violet staining provided a robust quantitative measure of biofilm inhibition, future studies using confocal laser scanning microscopy (CLSM) could provide deeper insights into the structural changes of the biofilm architecture under extract treatment.

Overall, these findings support continued investigation of *C. ovina*, particularly its ethanolic extract, as a potential source of bioactive compounds with antibacterial, antibiofilm, and virulence‐transcript–reducing activity against *S. saprophyticus*.

## 5. Conclusion

*C. ovina* extracts, particularly the ethanolic extract, demonstrated in vitro growth‐inhibitory and antibiofilm activity against clinical *S. saprophyticus* isolates and were associated with reduced *ureC* and *uafA* transcript levels in a MDR isolate. These results support further investigation, including chemical characterization, sub‐MIC antivirulence testing, safety assessment, and in vivo validation before clinical translation.

## Funding

No funding was received for this manuscript.

## Conflicts of Interest

The authors declare no conflicts of interest.

## Supporting information


**Supporting Information** Additional supporting information can be found online in the Supporting Information section. Supporting figures including determination of the MIC of aqueous and ethanolic extracts of *C. ovina* against SS1–SS3 isolates using the broth microdilution method, and biofilm inhibition of SS1–SS3 isolates treated with ethanolic and aqueous extracts.

## Data Availability

The data that support the findings of this study are available from the corresponding author upon reasonable request.
