# Supplementary material for: In Vitro Effects of Centaurea ovina (Roudbari Cornflower) Aqueous and Ethanolic Extracts on Staphylococcus saprophyticus: Growth Inhibition, Antibiofilm Activity, and Reduced ureC/uafA Transcripts
Source: Int J Microbiol. 2026 Jul 15;2026:7908693. doi: 10.1155/ijm/7908693 (PMC13370201; doi:10.1155/ijm/7908693)
Supplement: Supplementary file 1 — Supporting Information Additional supporting information can be found online in the Supporting Information section. Supporting figures including determination of the MIC of aqueous and ethanolic extracts of C. ovina against SS1–SS3 isolates using the broth microdilution method, and biofilm inhibition of SS1–SS3 isolates treated with ethanolic and aqueous extracts. [file IJM-2026-7908693-s001.docx]

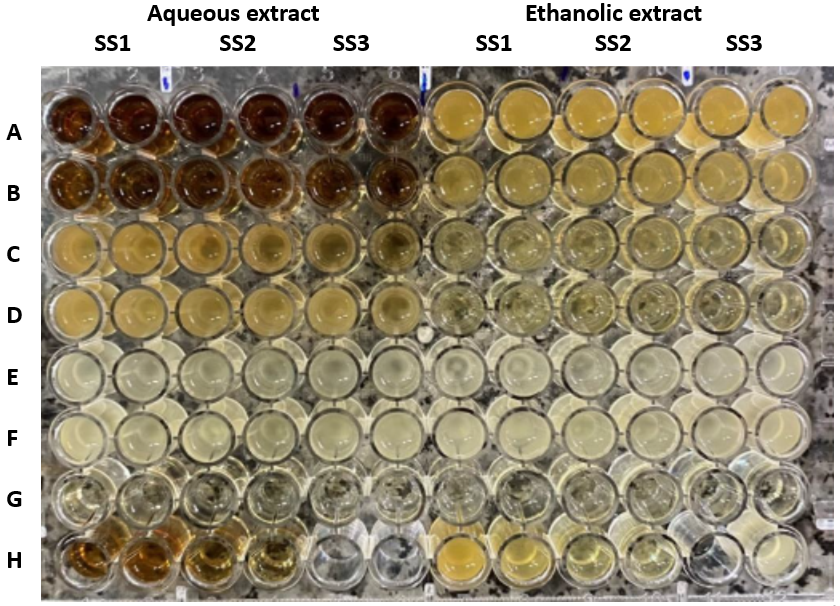


Determination of the MIC of aqueous and ethanolic extracts of *C. ovina* against SS1–SS3 isolates using the broth microdilution method. The columns corresponding to the aqueous extract (left panel) and ethanolic extract (right panel) were tested against three isolates (SS1–SS3). Rows A–D represent decreasing extract concentrations (48, 24, 12, and 6 mg/mL, respectively). Row E represents solvent controls (MHB + bacterial suspension with sterile distilled water for aqueous extract wells and DMSO for ethanolic extract wells). Row F represents the growth control (MHB + bacterial suspension without extract). Row G represents the sterility control (MHB only). Row H represents extract background/sterility controls (MHB + extract without bacterial inoculum).

|  | **Ethanolic extract** | | | **Aqueous extract** | | |
| --- | --- | --- | --- | --- | --- | --- |
|  | SS1 | SS2 | SS3 | SS1 | SS2 | SS3 |
|  | 80 | 75 | 60 | 60 | 50 | 40 |
|  |  |  |  |  |  |  |

|  | **Ethanolic extract** | | | **Aqueous extract** | | |
| --- | --- | --- | --- | --- | --- | --- |
|  | SS1 | SS2 | SS3 | SS1 | SS2 | SS3 |
|  | 80 | 75 | 60 | 60 | 50 | 40 |
|  |  |  |  |  |  |  |

Biofilm inhibition of SS1-SS3 isolates treated with ethanolic and aqueous extracts. The percentages represent the degree of biofilm inhibition observed for each bacterial isolate in response to the extracts.
